# (2*R*)-2-Benzene­sulfonamido-2-phenyl­ethanoic acid

**DOI:** 10.1107/S1600536809011611

**Published:** 2009-04-02

**Authors:** Muhammad Nadeem Arshad, M. Nawaz Tahir, Islam Ullah Khan, Muhammad Shafiq, Sarfraz Ahmad

**Affiliations:** a Department of Chemistry, Government College University, Lahore, Pakistan; b Department of Physics, University of Sargodha, Sargodha, Pakistan; c Pharmagen Ltd, Lahore 54000, Pakistan

## Abstract

In the title compound, C_14_H_13_NO_4_S, the dihedral angle between the aromatic ring planes is 45.52 (18)°. In the crystal structure, inter­molecular N—H⋯O and O—H⋯O hydrogen bonds lead to chains of mol­ecules propagating in [100] in which the the ring motifs *R*_2_^1^(8), *R*_2_^2^(8) and *R*_3_^3^(11) are apparent. These polymeric chains are linked through C—H⋯O inter­actions.

## Related literature

For related structures, see: Chaudhuri (1984 ▶); Shan & Huang (1999 ▶). For background, see: Arshad *et al.* (2008 ▶); Cama *et al.* (2003 ▶); Dankwardt *et al.* (2002 ▶); Zhi-jian *et al.* (2006 ▶). For graph-set notation, see: Bernstein *et al.* (1995 ▶).
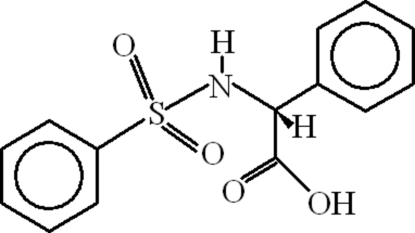


## Experimental

### 

#### Crystal data

C_14_H_13_NO_4_S
*M* *_r_* = 291.31
Orthorhombic, 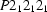

*a* = 5.6022 (5) Å
*b* = 12.5026 (9) Å
*c* = 19.7886 (15) Å
*V* = 1386.03 (19) Å^3^
*Z* = 4
Mo *K*α radiation
μ = 0.25 mm^−1^
*T* = 296 K
0.22 × 0.18 × 0.15 mm

#### Data collection

Bruker Kappa APEXII CCD diffractometer
Absorption correction: multi-scan (*SADABS*; Bruker, 2005 ▶) *T*_min_ = 0.944, *T*_max_ = 0.966
8476 measured reflections
2818 independent reflections
1679 reflections with *I* > 2σ(*I*)
*R*_int_ = 0.050

#### Refinement

*R*[*F* ^2^ > 2σ(*F* ^2^)] = 0.051
*wR*(*F* ^2^) = 0.091
*S* = 1.01
2818 reflections
182 parameters
H-atom parameters constrained
Δρ_max_ = 0.23 e Å^−3^
Δρ_min_ = −0.28 e Å^−3^
Absolute structure: Flack (1983 ▶), 1092 Friedal Pairs
Flack parameter: 0.02 (10)

### 

Data collection: *APEX2* (Bruker, 2007 ▶); cell refinement: *SAINT* (Bruker, 2007 ▶); data reduction: *SAINT*; program(s) used to solve structure: *SHELXS97* (Sheldrick, 2008 ▶); program(s) used to refine structure: *SHELXL97* (Sheldrick, 2008 ▶); molecular graphics: *ORTEP-3 for Windows* (Farrugia, 1997 ▶) and *PLATON* (Spek, 2009 ▶); software used to prepare material for publication: *WinGX* (Farrugia, 1999 ▶) and *PLATON*.

## Supplementary Material

Crystal structure: contains datablocks global, I. DOI: 10.1107/S1600536809011611/hb2938sup1.cif

Structure factors: contains datablocks I. DOI: 10.1107/S1600536809011611/hb2938Isup2.hkl

Additional supplementary materials:  crystallographic information; 3D view; checkCIF report

## Figures and Tables

**Table 1:** Hydrogen-bond geometry (Å, °)

*D*—H⋯*A*	*D*—H	H⋯*A*	*D*⋯*A*	*D*—H⋯*A*
N1—H1⋯O3^i^	0.86	2.55	3.155 (4)	128
O3—H3*O*⋯O4^ii^	0.82	1.83	2.646 (3)	176
C2—H2⋯O1^iii^	0.93	2.56	3.340 (5)	142
C3—H3⋯O2^iv^	0.93	2.54	3.225 (5)	131
C7—H7⋯O1^iii^	0.98	2.55	3.432 (4)	150

Symmetry codes: (i) 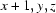; (ii) 
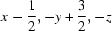
; (iii) 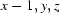; (iv) 
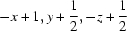
.

**Fig. 1.**
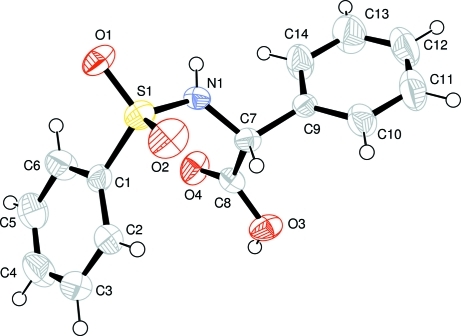
View of (I) with displacement ellipsoids drawn at the 50% probability level. H-atoms are shown by small circles of arbitrary radius.

**Fig. 2.**
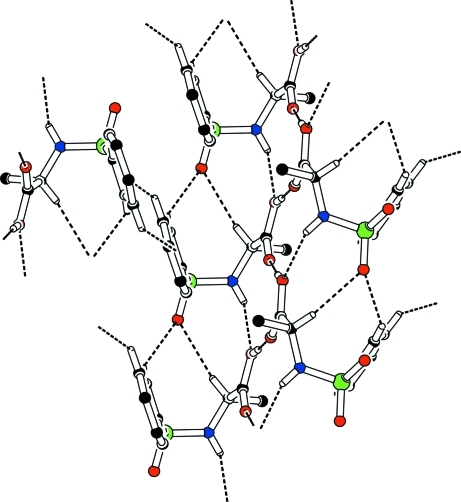
The partial packing of (I) showing that molecules form ring motifs through intermolecular H-bonding. The H-atoms not involved in H-bonding and the benzene ring of phenyl glycine are omited for clarity.

## supplementary crystallographic information

### Comment

Amino acid derived sulfonamides have been synthesized as ligands (Zhi-jian *et al.*, 2006) which showed potent procollagen C-proteinase (PCP) inhibition (Dankwardt *et al.*, 2002) and arginase inhibition (Cama *et al.*, 2003). The title compound (I), (Fig 1), has been prepared as an intermediate for the synthesis of our ongoing studies of thiazine (Arshad *et al.*, 2008) related heterocycles.

The crystal structure of (II) *R*-(-)-*N*-benzenesulfonylglutamic acid (Shan & Huang, 1999) and (III) *N*-Benzenesulfonyl-DL-alanine (Chaudhuri, 1984) have been published. These structures have a common group with (I) except the benzene ring of phenyl glycine.

The title compound has a chairal center at C7 with slightly distorted tetrahedral geometry with H7 at the apical position. The coordination around the S-atom is distorted tetrahedral. The molecules of the compound are stabilized due to strong intermolecular H-bonding (Table 1). Three ring motifs *R*_2_^1^(8), *R*_2_^2^(8) and *R*_3_^3^(11) (Bernstein *et al.*, 1995) are formed due to two H-bonds of C—H···O type, H-bonding of types C—H···O and N—H···O, and two O—H···O and N—H···O, respectively (Fig 2). The ring motifs are connected to each other in such a way that a rod-shaped attachement of molecules exist along the *a* axis. These polymeric chains are linked through the remaing H-bonding of C—H···O type.

### Experimental

Phenyl glycine (1 g, 6.6 mmol) was dissolved in distilled water (10 ml) in a round bottom flask (25 ml). The pH of the solution was maintained at 8–9 using 1*M*, Na_2_CO_3_ solution. Benzene sulfonyl chloride (1.16 g, 6.6 mmol) was then added to the solution and stirred at room temperature until all the benzene sulfonyl chloride was consumed. On completion of the reaction the pH was adjusted 1–2, using 1 N HCl. The precipitate obtained was filtered, washed with distilled water, dried and recrystalized in dichloromethane and methanol to yield colourless prisms of (I).

### Refinement

The H-atoms were positioned geometrically, with O—H = 0.82 Å N—H = 0.86 Å and C—H = 0.93–0.98 Å for aromatic, C-H = 0.98 Å and refined as riding with U_iso_(H) = 1.2U_eq_(carrier).

### Crystal data

**Table:**

C_14_H_13_NO_4_S	*F*(000) = 608
*M**_r_* = 291.31	*D*_x_ = 1.396 Mg m^−^^3^
Orthorhombic, *P*2_1_2_1_2_1_	Mo *K*α radiation, λ = 0.71073 Å
Hall symbol: P 2ac 2ab	Cell parameters from 2818 reflections
*a* = 5.6022 (5) Å	θ = 2.6–26.7°
*b* = 12.5026 (9) Å	µ = 0.25 mm^−^^1^
*c* = 19.7886 (15) Å	*T* = 296 K
*V* = 1386.03 (19) Å^3^	Prism, colorless
*Z* = 4	0.22 × 0.18 × 0.15 mm

### Data collection

**Table:**

Bruker Kappa APEXII CCD diffractometer	2818 independent reflections
Radiation source: fine-focus sealed tube	1679 reflections with *I* > 2σ(*I*)
graphite	*R*_int_ = 0.050
Detector resolution: 7.80 pixels mm^-1^	θ_max_ = 26.7°, θ_min_ = 2.6°
ω scans	*h* = −7→3
Absorption correction: multi-scan (*SADABS*; Bruker, 2005)	*k* = −15→15
*T*_min_ = 0.944, *T*_max_ = 0.966	*l* = −25→25
8476 measured reflections	

### Refinement

**Table:**

Refinement on *F*^2^	Secondary atom site location: difference Fourier map
Least-squares matrix: full	Hydrogen site location: inferred from neighbouring sites
*R*[*F*^2^ > 2σ(*F*^2^)] = 0.051	H-atom parameters constrained
*wR*(*F*^2^) = 0.091	*w* = 1/[σ^2^(*F*_o_^2^) + (0.0304*P*)^2^] where *P* = (*F*_o_^2^ + 2*F*_c_^2^)/3
*S* = 1.01	(Δ/σ)_max_ < 0.001
2818 reflections	Δρ_max_ = 0.23 e Å^−^^3^
182 parameters	Δρ_min_ = −0.28 e Å^−^^3^
0 restraints	Absolute structure: Flack (1983), 1092 Friedal Pairs
Primary atom site location: structure-invariant direct methods	Flack parameter: 0.02 (10)

### Special details

**Table:**

Geometry. Bond distances, angles *etc*. have been calculated using the rounded fractional coordinates. All su's are estimated from the variances of the (full) variance-covariance matrix. The cell e.s.d.'s are taken into account in the estimation of distances, angles and torsion angles
Refinement. Refinement of *F*^2^ against ALL reflections. The weighted *R*-factor *wR* and goodness of fit *S* are based on *F*^2^, conventional *R*-factors *R* are based on *F*, with *F* set to zero for negative *F*^2^. The threshold expression of *F*^2^ > σ(*F*^2^) is used only for calculating *R*-factors(gt) *etc*. and is not relevant to the choice of reflections for refinement. *R*-factors based on *F*^2^ are statistically about twice as large as those based on *F*, and *R*- factors based on ALL data will be even larger.

### Fractional atomic coordinates and isotropic or equivalent isotropic displacement parameters (Å^2^)

**Table:**

	*x*	*y*	*z*	*U*_iso_*/*U*_eq_	
S1	0.8906 (2)	0.51683 (6)	0.15187 (4)	0.0387 (3)	
O1	1.1438 (4)	0.50754 (15)	0.15986 (10)	0.0489 (9)	
O2	0.7303 (5)	0.44742 (17)	0.18723 (11)	0.0554 (9)	
O3	0.2940 (5)	0.62039 (15)	0.01637 (12)	0.0456 (10)	
O4	0.6710 (5)	0.68015 (15)	0.01169 (12)	0.0496 (9)	
N1	0.8409 (5)	0.49873 (16)	0.07161 (11)	0.0338 (9)	
C1	0.8088 (7)	0.6505 (2)	0.17079 (15)	0.0363 (13)	
C2	0.5942 (8)	0.6698 (3)	0.20125 (16)	0.0533 (14)	
C3	0.5343 (9)	0.7746 (4)	0.21780 (18)	0.0723 (19)	
C4	0.6886 (11)	0.8565 (3)	0.2012 (2)	0.075 (2)	
C5	0.8993 (9)	0.8359 (3)	0.16954 (19)	0.0640 (18)	
C6	0.9639 (7)	0.7318 (2)	0.15472 (16)	0.0521 (14)	
C7	0.6001 (7)	0.49830 (19)	0.04431 (13)	0.0316 (12)	
C8	0.5281 (8)	0.6104 (2)	0.02287 (14)	0.0340 (13)	
C9	0.5829 (7)	0.4265 (2)	−0.01808 (14)	0.0318 (13)	
C10	0.3966 (8)	0.3565 (2)	−0.02540 (17)	0.0507 (13)	
C11	0.3774 (10)	0.2936 (3)	−0.0832 (2)	0.0690 (18)	
C12	0.5456 (10)	0.3020 (3)	−0.1324 (2)	0.072 (2)	
C13	0.7293 (9)	0.3721 (3)	−0.1256 (2)	0.080 (2)	
C14	0.7496 (7)	0.4351 (3)	−0.06873 (18)	0.0583 (16)	
H1	0.95974	0.48921	0.04478	0.0406*	
H2	0.49001	0.61383	0.21076	0.0640*	
H3	0.39147	0.78931	0.23985	0.0865*	
H3O	0.26138	0.68249	0.00676	0.0547*	
H4	0.64818	0.92669	0.21182	0.0899*	
H5	1.00004	0.89197	0.15781	0.0768*	
H6	1.10972	0.71706	0.13426	0.0624*	
H7	0.48902	0.47247	0.07892	0.0379*	
H10	0.28216	0.35094	0.00847	0.0608*	
H11	0.25062	0.24614	−0.08804	0.0827*	
H12	0.53489	0.25964	−0.17091	0.0872*	
H13	0.84273	0.37763	−0.15968	0.0956*	
H14	0.87550	0.48313	−0.06466	0.0702*	

### Atomic displacement parameters (Å^2^)

**Table:**

	*U*^11^	*U*^22^	*U*^33^	*U*^12^	*U*^13^	*U*^23^
S1	0.0371 (7)	0.0395 (4)	0.0395 (4)	0.0025 (5)	−0.0015 (5)	0.0072 (4)
O1	0.0308 (18)	0.0569 (13)	0.0590 (14)	0.0051 (14)	−0.0105 (13)	0.0060 (12)
O2	0.055 (2)	0.0573 (14)	0.0538 (14)	−0.0093 (13)	0.0066 (13)	0.0230 (11)
O3	0.037 (2)	0.0322 (13)	0.0677 (16)	0.0072 (13)	0.0039 (16)	0.0136 (12)
O4	0.048 (2)	0.0318 (11)	0.0690 (16)	−0.0128 (14)	−0.0048 (14)	0.0143 (10)
N1	0.030 (2)	0.0351 (13)	0.0364 (13)	0.0025 (15)	0.0059 (13)	−0.0056 (11)
C1	0.031 (3)	0.0481 (19)	0.0297 (18)	−0.0040 (18)	0.0001 (17)	−0.0046 (14)
C2	0.045 (3)	0.067 (2)	0.048 (2)	0.001 (3)	0.000 (2)	−0.0200 (17)
C3	0.049 (4)	0.096 (3)	0.072 (3)	0.016 (3)	−0.006 (2)	−0.039 (3)
C4	0.085 (5)	0.059 (3)	0.082 (3)	0.015 (3)	−0.011 (3)	−0.033 (2)
C5	0.070 (4)	0.046 (2)	0.076 (3)	−0.010 (2)	−0.004 (3)	−0.0124 (19)
C6	0.057 (3)	0.0494 (19)	0.050 (2)	0.001 (2)	0.009 (2)	−0.0060 (19)
C7	0.032 (3)	0.0251 (15)	0.0378 (16)	0.0002 (19)	0.0039 (17)	0.0037 (12)
C8	0.041 (3)	0.0298 (18)	0.0311 (17)	0.002 (2)	0.003 (2)	−0.0001 (14)
C9	0.030 (3)	0.0242 (14)	0.0412 (18)	0.0008 (17)	0.0001 (18)	0.0012 (13)
C10	0.057 (3)	0.0360 (17)	0.059 (2)	−0.006 (2)	0.003 (2)	−0.0045 (16)
C11	0.082 (4)	0.045 (2)	0.080 (3)	−0.014 (2)	−0.017 (3)	−0.012 (2)
C12	0.093 (5)	0.062 (3)	0.062 (3)	−0.005 (3)	−0.011 (3)	−0.027 (2)
C13	0.078 (5)	0.103 (3)	0.059 (3)	−0.017 (3)	0.020 (3)	−0.029 (2)
C14	0.059 (4)	0.068 (2)	0.048 (2)	−0.022 (2)	0.013 (2)	−0.017 (2)

### Geometric parameters (Å, °)

**Table:**

S1—O1	1.432 (3)	C9—C14	1.374 (5)
S1—O2	1.432 (3)	C9—C10	1.370 (5)
S1—N1	1.628 (2)	C10—C11	1.392 (5)
S1—C1	1.773 (3)	C11—C12	1.359 (7)
O3—C8	1.324 (5)	C12—C13	1.358 (7)
O4—C8	1.204 (4)	C13—C14	1.378 (5)
O3—H3O	0.8200	C2—H2	0.9300
N1—C7	1.453 (5)	C3—H3	0.9300
N1—H1	0.8600	C4—H4	0.9300
C1—C2	1.366 (6)	C5—H5	0.9300
C1—C6	1.375 (4)	C6—H6	0.9300
C2—C3	1.392 (6)	C7—H7	0.9800
C3—C4	1.380 (7)	C10—H10	0.9300
C4—C5	1.361 (7)	C11—H11	0.9300
C5—C6	1.382 (5)	C12—H12	0.9300
C7—C8	1.519 (4)	C13—H13	0.9300
C7—C9	1.530 (4)	C14—H14	0.9300
			
O1—S1—O2	121.22 (14)	C10—C11—C12	119.4 (4)
O1—S1—N1	105.42 (14)	C11—C12—C13	120.3 (4)
O1—S1—C1	108.01 (15)	C12—C13—C14	120.8 (4)
O2—S1—N1	106.58 (14)	C9—C14—C13	119.7 (4)
O2—S1—C1	107.82 (16)	C1—C2—H2	121.00
N1—S1—C1	107.03 (13)	C3—C2—H2	121.00
C8—O3—H3O	109.00	C2—C3—H3	120.00
S1—N1—C7	121.5 (2)	C4—C3—H3	120.00
S1—N1—H1	119.00	C3—C4—H4	120.00
C7—N1—H1	119.00	C5—C4—H4	120.00
C2—C1—C6	121.9 (3)	C4—C5—H5	120.00
S1—C1—C2	119.2 (3)	C6—C5—H5	120.00
S1—C1—C6	119.0 (3)	C1—C6—H6	121.00
C1—C2—C3	118.8 (4)	C5—C6—H6	121.00
C2—C3—C4	119.5 (4)	N1—C7—H7	109.00
C3—C4—C5	120.8 (4)	C8—C7—H7	109.00
C4—C5—C6	120.2 (4)	C9—C7—H7	109.00
C1—C6—C5	118.8 (4)	C9—C10—H10	120.00
C8—C7—C9	107.4 (2)	C11—C10—H10	120.00
N1—C7—C9	111.2 (3)	C10—C11—H11	120.00
N1—C7—C8	110.3 (3)	C12—C11—H11	120.00
O3—C8—O4	124.9 (3)	C11—C12—H12	120.00
O3—C8—C7	112.2 (3)	C13—C12—H12	120.00
O4—C8—C7	122.9 (4)	C12—C13—H13	120.00
C7—C9—C10	120.6 (3)	C14—C13—H13	120.00
C7—C9—C14	120.0 (3)	C9—C14—H14	120.00
C10—C9—C14	119.4 (3)	C13—C14—H14	120.00
C9—C10—C11	120.4 (4)		
			
O1—S1—N1—C7	177.57 (18)	C4—C5—C6—C1	1.8 (6)
O2—S1—N1—C7	47.6 (2)	N1—C7—C8—O3	−161.7 (2)
C1—S1—N1—C7	−67.6 (2)	N1—C7—C8—O4	20.1 (4)
O1—S1—C1—C2	−145.8 (3)	C9—C7—C8—O3	77.0 (3)
O1—S1—C1—C6	33.9 (3)	C9—C7—C8—O4	−101.2 (4)
O2—S1—C1—C2	−13.2 (3)	N1—C7—C9—C10	134.8 (3)
O2—S1—C1—C6	166.5 (3)	N1—C7—C9—C14	−48.0 (4)
N1—S1—C1—C2	101.2 (3)	C8—C7—C9—C10	−104.5 (3)
N1—S1—C1—C6	−79.2 (3)	C8—C7—C9—C14	72.7 (4)
S1—N1—C7—C8	90.6 (2)	C7—C9—C10—C11	178.0 (3)
S1—N1—C7—C9	−150.35 (18)	C14—C9—C10—C11	0.8 (5)
S1—C1—C2—C3	178.1 (3)	C7—C9—C14—C13	−178.3 (3)
C6—C1—C2—C3	−1.5 (5)	C10—C9—C14—C13	−1.0 (5)
S1—C1—C6—C5	180.0 (3)	C9—C10—C11—C12	0.1 (6)
C2—C1—C6—C5	−0.4 (5)	C10—C11—C12—C13	−0.8 (7)
C1—C2—C3—C4	2.1 (6)	C11—C12—C13—C14	0.5 (7)
C2—C3—C4—C5	−0.7 (6)	C12—C13—C14—C9	0.4 (6)
C3—C4—C5—C6	−1.3 (6)		

### Hydrogen-bond geometry (Å, °)

**Table:**

*D*—H···*A*	*D*—H	H···*A*	*D*···*A*	*D*—H···*A*
N1—H1···O3^i^	0.86	2.55	3.155 (4)	128
O3—H3O···O4^ii^	0.82	1.83	2.646 (3)	176
C2—H2···O1^iii^	0.93	2.56	3.340 (5)	142
C3—H3···O2^iv^	0.93	2.54	3.225 (5)	131
C7—H7···O1^iii^	0.98	2.55	3.432 (4)	150

Symmetry codes: (i) *x*+1, *y*, *z*; (ii) *x*−1/2, −*y*+3/2, −*z*; (iii) *x*−1, *y*, *z*; (iv) −*x*+1, *y*+1/2, −*z*+1/2.
